# Editorial: Management of the hypoplastic left heart syndrome: from fetus to adult

**DOI:** 10.3389/fped.2023.1232445

**Published:** 2023-06-13

**Authors:** Milan Prsa, Heynric B. Grotenhuis, Otto Rahkonen

**Affiliations:** ^1^ Woman-Mother-Child Department, Lausanne University Hospital and University of Lausanne, Lausanne, Switzerland; ^2^ Department of Pediatric Cardiology, Utrecht Medical Center, Utrecht, Netherlands; ^3^ Department of Pediatric Cardiology, New Children's Hospital, Helsinki University Hospital, Helsinki, Finland

**Keywords:** hypoplastic left heart syndrome, fetal interventions, neurocognitive outcome, tricuspid valve regurgitation, fontan associated liver disease, lymphatic insufficiency, fontan failure

**Editorial on the Research Topic** Management of the hypoplastic left heart syndrome: from fetus to adult

Hypoplastic left heart syndrome (HLHS) is one of the most complex forms of congenital heart disease (CHD). It can be defined as a spectrum of cardiac malformations featuring significant degrees of underdevelopment of left heart structures (mitral valve, left ventricle, aortic valve, ascending aorta, and aortic arch), which are unable to provide systemic perfusion. Considered uniformly fatal a few decades ago, extraordinary efforts have been invested in developing interventions that bring the promise of long-term survival, resulting in an increasing number of children and adults living with HLHS today. However, management of HLHS remains exceptionally challenging, compelling physicians to build considerable knowledge and expertise, while expending inordinate amounts of energy and resources that are incommensurate with the prevalence of this cardiac malformation.

Currently, the most common strategy for survival involves a series of palliative surgical interventions, performed in three stages. The first stage Norwood procedure, performed soon after birth, consists of recruiting the right ventricle to provide systemic output through a reconstructed aorta and aortic arch, excising the atrial septum, and placing a systemic-to-pulmonary artery shunt (Blalock-Taussig-Thomas shunt) or a right ventricle-to-pulmonary artery conduit (Sano shunt) to deliver pulmonary blood flow. The second stage, performed around 3 to 6 months of age, involves taking down the systemic-to-pulmonary shunt, and connecting the superior vena cava to the pulmonary arteries (bidirectional Glenn anastomosis), while the third stage, performed at 2 to 4 years of age, redirects the inferior vena cava to the pulmonary arteries (Fontan operation), completing a total cavopulmonary connection. The result is an abnormal circulation where a single right ventricle pumps blood to the systemic circulation and the systemic venous return flows passively to the pulmonary arteries.

Since the first successful Norwood procedure reported in 1983 (1), significant strides have been made in improving diagnosis and preoperative care, refining surgical techniques and anaesthesia, and advancing postoperative management of patients with HLHS, with outcome data now available for over 30 years (2). Although survival has improved significantly, mortality after the neonatal Norwood procedure has plateaued around 10%–15% in the most experienced centers, and interstage mortality remains elusively high, with pre-Glenn and pre-Fontan attrition rates both around 10% (2–5). Longer term survival without transplant is thus at best 60%–70% in the current era, making HLHS one of the most pernicious forms of CHD (6). For those who do survive beyond the three stages of surgery, the promise of longevity and a reasonably normal quality of life is still far out of reach. Most patients will face long-term complications such as neurocognitive impairment, atrioventricular valve regurgitation and Fontan-associated liver disease, while some will suffer the devastating and life-threatening complications of plastic bronchitis and protein-losing enteropathy. As the unsustainable Fontan physiology starts to decline in the second and third decades of life, circulatory failure and the prospect of heart transplant become a reality for almost all survivors.

Nevertheless, we continue undeterred in our pursuit to improve the quality and duration of life of patients with HLHS, and we have witnessed impressive new advances in medical and surgical management since the new millennium. Prenatal diagnosis is allowing thorough prenatal counselling and fetal interventions, hybrid procedures and staged left ventricular recruitment strategies are offering new and promising treatment options, while new and improved imaging techniques stand to facilitate timely diagnosis and management of long-term complications.

This Research Topic delivers a series of review articles aiming to address some of the most important pitfalls in the current management strategies for the spectrum of HLHS. Starting with a review of the role and potential benefits of fetal interventions and maternal hyperoxygenation on the evolving or established HLHS, Tulzer et al. showcase how advances in fetal echocardiography gave rise to the opportunity to better understand and maybe even modify the natural history of the disease prior to birth. Moving to a discussion of long-term complications, Knirsch et al. perform an exhaustive review of the literature analyzing the neurodevelopmental as well as functional outcomes after stage 1 hybrid palliation and how they compare to the classic Norwood procedure, while de Lange et al. provide an in-depth review of how to diagnose and manage Fontan-associated liver disease, calling attention to the role of advanced imaging and surveillance protocols in staving off its inevitable progression. A further challenge in the management of HLHS is the regurgitation of the tricuspid valve, which is associated with a poor prognosis and whose successful repair improves outcome (7, 8). Bharucha and Viola highlight the insights provided by echocardiography in understanding and recognizing the mechanisms of tricuspid valve dysfunction specific to HLHS, which can be of critical importance when considering the appropriate timing and surgical techniques for repair. Special attention is also devoted to the abnormal lymphatic circulation and its devastating consequences in single ventricle physiology. Hanser et al. demonstrate that severe lymphatic abnormalities correlate with reduced exercise capacity and symptoms of Fontan failure in the long-term follow-up of patients with total cavopulmonary connection, while Bauer et al. discuss current and emerging strategies to diagnose and treat lymphatic insufficiency in HLHS. Lastly, in an excellent review of circulatory failure after Fontan surgery facing all patients with HLHS, Kamsheh et al. encapsulate the mechanisms underlying the inherent unsustainability of the Fontan circulation and make a compelling case for a pressing need to improve the management and outcomes of patients with a failing Fontan circulation.

The guest editors are grateful to the international experts in the field who have contributed to this Research Topic, the reviewers for their critical appraisal, and thank the readers for their interest in the subject.
